# Dr. Charles McBurney

**Published:** 1913-12

**Authors:** 


Dr. Charles McBurney, P. & S. of N. Y., 1870, the well known New York Surgeon, died at Brookline, Mass., November 7, suddenly, probably from the strain of a hunting trip. He was born at Roxbury, Mass., February 17, 1845, and was educated at the

Roxbury Latin School and Harvard University, from which he received the degree of A. B. in 1866, and of A. M., a few years later. Of his many honors and his notable achievements in surgery, especially in that of the appendix, we need not speak at length. Recently he had resided in Stockbridge, Mass., though retaining an office in New York.

